# The Treatment of Acute Gonorrhoea

**Published:** 1911-10

**Authors:** Charles W. Bethune

**Affiliations:** 131 Allen Street, Buffalo, N. Y.


### BUFFALO MEDICAL JOURNAL

VOL. LXVII. OCTOBER, 1911. NO. 3

### ORIGINAL COMMUNICATIONS

### The Treatment of Acute Gonorrhoea

By CHARLES W. BETHUNE, M.D.
131 Allen Street, Buffalo, N. Y.

IN the treatment of gonorrhoea, as well as other infections, if one's efforts are rewarded by a complete and rapid recovery, too much credit is apt to be ascribed to the treatment and too little to the patient's individual resistance. The relation of the individual resistance of the patient to the individual virulence of the infecting organism is the most important factor in every infectious disease. All that one can hope to accomplish through treatment is to turn the balance in the patient's favor by increasing his immunity and diminishing the virulence of the infection.

During coitus the friction of the vaginal walls upon the lateral surfaces of the glans causes the meatus to gape open, facilitating the entrance of infectious material. Most of this is washed out by the first subsequent urination and were it not for the fact that some of the gonococci may already have entered the numerous tiny intercellular spaces, or that the lacuna magna is so constructed that it cannot be flushed out by the urinary stream, gonorrhoea would be a rare disease.

In addition to these mechanical factors, it must be remembered that some individuals, although notorious rakes, are blessed with such a strong natural immunity that they never become infected. Urination immediately after coitus followed by the instillation of a few drops of a freshly prepared solution of some organic silver salt, Argyrol 5-10 per cent. Protargol 1-3 per cent., is of unquestionable prophylactic value. Irrigation of the penile urethra by the Janet method with potassium permanganate 1-5,000, care being taken to flush the lacuna by directing the stream towards the roof of the fossa, is almost invariably successful if employed within 24 hours after coitus.

The onset of gonorrhoea is heralded by a slight tingling in the fossa navicularis, which draws the patient's attention to his penis. He usually seeks seclusion and proceeds to inspect the organ. The lips of the meatus are found to be slightly swollen and reddened and a drop of muco-pus may be expressed. Medical advice is seldom sought during this stage, but various quack remedies are tried until after two or three days the increasing burning urination, painful erections and copious discharge, drive him to the physician.

The history of the case should first be taken, notes being made of previous genito-urinary history, habits, date of last coitus, date of onset and presence of painful erections or chordee. It must be remembered that the last coitus may not have been the infecting one, so that the incubation period can only be calculated in cases where there has been no previous exposure for several weeks. The character and seat of pain, whether before, during, after or between urinations, should be ascertained. The penis, praepuce, inguinal nodes, testicles and cord should be inspected or palpated and any deviations from normal noted. A drop of the discharge should be smeared upon two cover slips and stained, one by methylene blue and the other by Gram's. This is of the utmost importance, in order to be sure that we are dealing with a case of gonorrhoea and not an infection by some other pyogenic organism, a urethral chancre or chancroid.

The patient then passes his urine into two large test tubes. If the first tube is cloudy and the second clear, the infection is limited to the anterior urethra; if both are cloudy, the posterior is also infected. A drop of nitric acid should always be added to it to eliminate cloudiness due to phosphates. The test tubes must always be boiled after each patient to eliminate all danger of accidental infection of the next user. This so-called two glass test, for differentiating anterior from posterior gonorrhoea, is of value only during the acute stage or when the discharge is profuse, for when the pus in the posterior urethra becomes too scanty to contaminate the urine in the bladder, the first jets of urine wash out all pus and the second tube is clear.

I have given all methods of treating acute gonorrhoea a thorough trial and have always returned to the Janet method of irrigating with potassium permanganate, finding that it relieves the pain and diminishes the discharge more quickly, and is followed by a smaller proportion of complications than any other method. After urinating, the patient washes all pus and smegma from the penis, drops his trousers to the knees, pulls his shirt up to the umbilicus and reclines upon the table in Valentine's position. Either an ordinary or the Wohlbarst G-U basin should be placed between the thighs to catch the return flow.

There are many tips for irrigating, each operator having a preference—I naturally for my own, illustrated later. In my tip the automatic spring cut-off enables one to regulate the force of the stream by a light pressure with the little finger, releasing the pressure shuts off the flow. A tight connection is assured by tying a string around the rubber tube over the groove in the handle of the tip. The tip is made by the Sands-Levy Co.

The reservoir of the irrigator is raised 18-24 inches above the top of the table and filled with a warm (105-110 F), 1-5,000 solution of potasium permanganate. The praepuce is retracted and the penis is grasped at the coronary sulcus with the thumb and index finger, the remaining fingers encircling the organ. The tip is lightly held by the spring handle, pressure of the little finger upon the finger-rest regulating the flow. Press upon the spring until the purple solution appears, then shut off the flow, hold the tip one-quarter inch from the meatus, press upon the spring and allow the fluid to flow gently into the meatus.

When the can is empty, drop the tip into the sterilizer, remove the basin, empty it, and dip the notched end into boiling water or pass it through the flame. Neglecting to sterilize the tip will result in infecting patients with more virulent strains of gonococci not to mention syphilis.

Hand the patient a gauze sponge, instructing him to dry the genitals. A three-inch square of gauze in the center of which is a slit three-quarters of an inch long is then pulled over the glans and the foreskin pulled down over it. This is to be changed at each urination. Circumcised patients should wear a gonorrhoeal bag, changing the cotton at each urination.

The patient is then told of the danger of infecting the eyes, is instructed to wash his hands after handling the genitals and to abstain from all alcoholic liquors. Sexual dalliance, coitus or the reading of erotic literature, is absolutely prohibited. Smoking in moderation is not considered harmful. No internal treatment is given as a routine measure beyond drinking as much milk and water as possible to dilute the urine, thus lowering its specific gravity and acidity. The frequent urination also serves to flush the irritating pus out of the urethra.

The various balsams, aromatic oils, and the like, at one time so popular in the treatment of gonorrhoea, are gradually being abandoned, as they have practically no effect upon the urethra, only serving to upset the stomach, thus reducing the patient's natural resistance to the infection.

Constipation is best relieved by cascara. All aloin preparations are to be avoided, as they increase pelvic congestion, markedly aggravating the patient's discomfort. Enemas administered by the patient are taboo, because of the danger of infecting the rectum. A well fitting suspensory relieves the dull, throbbing ache in the testicles and is a valuable prophylactic against epididymitis.

After the first irrigation the patient perceives that the dysuria is markedly diminished, but recommences within twenty-four hours. If the irrigation did no more than to relieve the excruciating pain accompanying micturition, its use would be indicated in every case. But when proper techinc is observed, its employment is also followed by a marked diminution of the discharge and a smaller proportion of complications than with any other treatment.

The irrigation treatment does not markedly alleviate the distressing painful, nocturnal erections or chordee. If the patient arises and urinates, the erection will subside; applications of cold water also give quick relief. Anaphrodisiac drugs are at the most very uncertain in action.

The irrigation must be repeated every twenty-four hours. On the second day the tip may be placed against the meatus and the can elevated three inches higher than on the preceding day. The elevation of the can may be increased two or three inches daily during the acute stage, provided the increased force of the stream does not cause pain. In no casė should the elevation exceed four feet.

Pain during or following the irrigation is a warning always to be heeded that the stream is too forcible. The most frequent cause of disaster during the acute stage is the ballooning out of the urethra by pressing the tip too firmly into the meatus, not to mention filling the bladder by forcing the cut off muscle. Failure to observe these two points has brought the method into disrepute with many surgeons.

If the patient has gone untreated for the first week or two, the infection will invariably have invaded the posterior urethra. Improper treatment, introduction of urethral instruments, irrigation with a catheter, rough and forcible Janet irrigations and neglecting to urinate immediately before local treatment, are also important factors in the etiology of posterior invasion. When properly conducted irrigations are instituted during the first week, a large proportion escape it.

The onset of posterior invasion is heralded by frequent urgent urination, diminution of the discharge, malaise, and cloudiness of both glasses of urine. The symptoms may vary in severity from very mild manifestations of those enumerated to an intense throbbing pain in the rectum and perinaeum, constant agonizing desire to urinate, and urination terminating with severe tenesmus, often accompanied by a terminal hematuria.

The onset of posterior involvement does not counterindicate the continuance of irrigations. The inflammation of the anterior urethra persists in spite of the diminution of the discharge and unless it is combated by the irrigations, it will break out afresh as soon as the posterior symptoms subside. The diminution of the discharge during the posterior stage has been the subject of much discussion, the most logical explanation being that the toxines are readily absorbed from the posterior urethra and stimulate the formation of antibodies, temporarily raising the immunity. Returning to the subject of irrigations, no attempt must be made during the acute posterior stage to force the cut-off muscle.

The milder forms of posterior urethritis require no special treatment beyond placing a hot water bag against the perinaeum at night to relieve the constant desire to urinate, or placing a pillow or two beneath the hips, which accomplishes the same result by relieving pelvic congestion. In the more severe cases a tablet may be administered at night containing Morphine, gr. ¼, combined with Atropine gr. 1-150. Hot rectal douches (110-120F) administered through a Kemp tip are also very soothing. I have found the following rectal suppository to be even more efficacious than either of the above methods.

| | |
|---|---|
| Ung. Crede | ʒss |
| Ichthyol | ʒj |
| Ol. Theobrom. Q. S. | |
| M. & div. in suppositoria No. vi | |
| Sig. Insert at night. | |

Massaging the acutely inflamed prostate often results in the expulsion of a tree-like cast of muco-purulent material followed by a sudden and complete abatement of the symptoms. Were it not for the fact that epididymitis follows in about 50 per cent. of all cases treated by this method, it would be the treatment par excellence. The vast majority of posterior infections subside spontaneously long before the anterior infection is cured. The subsidence of the acute posterior stage merges into the subacute stage which, together with the complications of gonorrhoea, will be discussed in subsequent papers.

131 Allen St.

Acknowledgments:
Valentine; Keyes; DeKeersmacher & Veerhogen; Vecki; and Bauman.

---

Doctor—If you want a medical book (see Mosby and Rebman advertisements), typewriter, vacuum cleaner, an instrument or almost anything else for your office or home, we can save you money and time. Telephone the editor before 1 P. M.

